# Toughness Behavior of SBR Acrylate Copolymer-Modified Pervious Concrete with Single-Sized Aggregates

**DOI:** 10.3390/ma14175089

**Published:** 2021-09-06

**Authors:** Chaohua Zhao, Hualin Li, Yi Peng, Xiaoyao Jia, Ali Rahman

**Affiliations:** 1 School of Civil Engineering, Chongqing Jiaotong University, Chongqing 400074, China; chaohua_zhao@163.com (C.Z.); hualin_li199508@163.com (H.L.); xiaoyaojxy@126.com (X.J.); 2 College of Traffic & Transportation, Chongqing Jiaotong University, Chongqing 400074, China; 3 Key Laboratory of Highway Engineering of Sichuan Province, Chengdu 610031, China; a.rahman@my.swjtu.edu.cn

**Keywords:** eco-efficient, pervious concrete, copolymer, toughness, sustainability

## Abstract

Pervious concrete is an eco-efficient concrete but has problems regarding its mechanical performance and permeability balance. This research investigated the feasibility of using a combination of styrene–butadiene rubber (SBR) and acrylate polymer to improve the toughness of pervious concrete while keeping its permeability. Single-sized aggregate and no sand were considered in the concrete mixture. Acrylate polymers with different solid content, PH, density, and viscosity were emulsion copolymerized with an SBR polymer. Eleven scenarios with different mix proportions and 220 specimens for compressive strength, flexural strength, flexural stiffness, impact resistance, and fracture toughness tests were selected to evaluate the effects of the copolymer on the toughness of copolymer-modified pervious concrete (CMPC). The studies showed that (1) the influence trend of the copolymers generally varied according to different mechanical indexes; (2) XG–6001 acrylate polymer mainly and comprehensively enhanced the toughness of the CMPC; (3) it was difficult to increase the enhancing property of the XG–6001 acrylate polymer with the growth of its mix proportion; (4) the zero-sand pervious concrete with 90% SBR and 10% XG–6001 acrylate emulsion copolymerization proved to have relatively high toughness. The proposed CMPC holds promising application value in sustainability traffic road construction.

## 1. Introduction

Pervious concrete has recently regained popularity as it is considered as an eco-efficient concrete in the sustainable development of transportation infrastructure [1]. Normal pervious concrete is mainly composed by virtue of large aggregates with little to no fine aggregates [2]. Many countries have adopted pervious concrete to infiltrate and purify the rainwater, to reduce traffic noise, and to raise the pavement skid resistance [3,4] due to its significant air void ratio and well-textured surface [5]. However, confined by the defects of easy cracking, pervious concrete is merely applied in traffic roads or parking lots subject to lower traffic load.

Polymers are added to the pervious concrete mixes, either in the form of an aqueous emulsion or in a dispersed form, in order to improve its mechanical behavior [2,6]. The polymer-modified pervious concrete (PMPC) has strong anti-cracking properties and durability; hence, many researchers have attempted to further enhance its performance and broaden its applications [3,7]. Styrene–butadiene rubber (SBR) polymer is widely used for its contribution to the modified physical and chemical properties of cement [8]. Kevern et al. [9] found that SBR enhanced the workability, strength, permeability, and freeze–thaw behavior, and it had a limited cement paste volume and high air void ratio. Huang et al. [10] revealed that the addition of SBR polymer latex could increase the tensile strength of pervious concrete, while sand decreased the porosity and permeability. Other researchers [6,11] provided an overview of PMPC with sand. They demonstrated that sand addition increases the mechanical properties (such as compressive strength and flexural strength), but its effect on PMPC is influenced by the water content, and thus low-void and permeability issues occur. Generally, the stability and durability of pervious concrete is strongly affected by compressive strength [12], and the American Concrete Institute argues that the pervious concrete with high porosity has limited application owing to its low compressive strength of 5–10 MPa [10,13]. Aoki et al. [14] pointed out that little significant difference existed between the mechanical properties of pervious concrete containing fly ash and those comprising only cement as a cementitious agent wherein its compressive strengthen was 10.06 MPa. Manan et al. [12] investigated PMPC’s mechanical performance by increasing sand content from zero to 100% and pointed out that the PMPC without sand had compressive strength of no more than 11.3 MPa. Despite this, they still recommended pervious concrete with low sand content for pavements of parking and walking areas owing to its high permeability.

However, Sofi et al. [15] reported that the incorporation of rubber particles leads to side effects on the compressive and flexural strengths of the concrete due to the poor bonding between cement paste and rubber particles and due to the softness of the rubber particles. Hence, many researchers addressed these issues by means of improving the polymer additives’ performance [16,17]; the copolymer was found to effectively modify pervious concrete wherein the porosity was increased, while a significant improvement in the later stage mechanical strength was observed [18]. Benyahia et al. [19] revealed that the emulsion copolymerization is a convenient, fast, and low-cost alternative to produce the copolymer by dispersing the polymer monomers in the droplets of an aqueous phase. Łaźniewska-Piekarczyk et al. [20] demonstrated that the combination of two different types of polycarboxyl ether by emulsion copolymerization could significantly affect the properties of fresh and hardened high-performance self-compacting concrete. Lang Lei et al. and Zhang Peng et al. [21,22] further demonstrated that the copolymer was capable of significantly improving concrete’s unconfined compressive strength and the strength development of cement-solidified dredged sludge. In addition, acrylate polymer was proved to endow grout with less toxicity, adjustable gelation time, low permeability, and high compressive strength, thus resulting in a wide application of acrylic grout in various fields, including dams, foundations, and tunnels [23]. Furthermore, acrylate was copolymerized with other materials, such acrylamide or styrene, to enhance grout’s mechanical, physical, and rheological properties [24]. Even so, little research has considered improving the mechanical properties of the pervious concrete mixture without sand, and research on the enhancement of acrylate copolymer to PMPC has not been sufficient.

Toughness is a measure of the energy absorption capacity of reinforced concrete and represents the concrete’s ability to resist fracture when subjected to static or dynamic load [25,26]. A series of serviceability-based toughness evaluating indexes is recommended by American standard ACI 544-2R, and the corresponding test methods, such as flexural strength test, drop-weight test, and fracture toughness test, are applicable to concretes reinforced with a polymer [27,28,29]. Mindess et al. [30] pointed out that toughness index values were of significance to specify and qualify control purposes for concrete mixture design. Murali et al. [31] estimated that a two-stage fiber-reinforced concrete benefited from higher fiber in terms of greater impact resistance properties. Sallal et al. [32] even modified the repeated drop-weight impact test for better locating the steel ball load distributer on the specimens and then obtaining a more stable toughness test value. Furthermore, Meng et al. [33] conducted four-bending tests for evaluating the flexural performance and toughness characteristics of a pervious concrete beam. The Chinese standards also specify toughness indexes with respect to polymer-reinforced concrete (e.g., flexural strength, flexural stiffness, and fracture toughness) [34,35]. It should be noted that the toughness of pervious concrete enhanced by polymers requires detailed research. Furthermore, there is still a need to understand how to obtain the desired polymerized pervious concrete with sufficient strength and toughness.

The aim of this study is to demonstrate the feasibility of using a combination of SBR and acrylate polymer to improve the toughness of CMPC with zero sand by means of a laboratory testing program and comprehensively statistical manners.

## 2. Materials and Methods

### 2.1. Materials

Single-sized pervious concrete has less shrinkage, high permeability, and noise elimination. This property was observed in our team’s earlier research, and a new, high mechanical performance porous cement pavement was further composed by enhancing its bonding paste with zero sand [36,37,38]. In this study, single-sized pervious concrete with zero sand was also chosen to investigate the toughness of CMPC. Portland cement, coarse aggregates, and multiple polymer latex were used to prepare the CMPC mixture. The characteristics of these materials are examined in the following sections.

#### 2.1.1. Cement

Portland cement with a strength grade of 42.5 (produced by Huaxin Cement Co., Ltd., Chongqing, China) was adopted. Its chemical compositions are presented in Table 1.

#### 2.1.2. Aggregate

**Figure 1:**
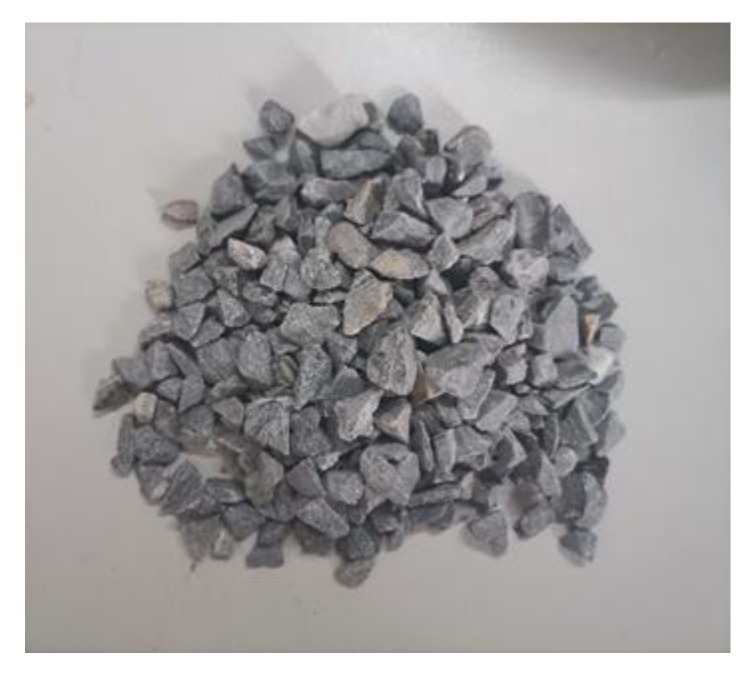
Image of aggregate sample.

Coarse aggregates of the pervious concrete were crushed limestone (mined in Chongqing) ranging from 4.75 to 9.5 mm in size, in which the nominal maximum aggregate size was 10 mm. After washing, the aggregates were oven dried at 120 °C. The clean and dry aggregates are shown in Figure 1.

#### 2.1.3. Polymer Latex Preparation

In this study, the copolymer latex was prepared by emulsion copolymerization of SBR and acrylate polymer [39]. The three types of the acrylate polymer latex were XG–6161 acrylate (further identified as P1), XG–6001 acrylate (further identified as P2), and XG–2135 acrylate (further identified as P3). The main characteristics of the polymers are shown in Table 2.

### 2.2. Mix Design and Sample Preparation

The basic mix proportions of the pervious concrete mixture used in this study were water, cement, and aggregate in a ratio of 0.41:1:4.7 by weight according to our team’s previous work [36,40,41]. Combinations of polymers were considered to enhance the mechanical performance of the pervious concrete; thus, P1–P3 were each selected to be mixed with SBR to investigate the effects of copolymers on pervious concrete. In addition, the water was replaced by the copolymer latex. In the copolymer latex, the mix proportions of 10%, 20%, and 30% for acrylate polymer (e.g., P1, P2, and P3) in the latex were used; hence, 11 scenarios of the copolymer latex were selected to prepare samples and investigate the effects of the copolymer on the performance and properties of the pervious concrete. The mix proportion of SBR to acrylate polymer for each scenario is shown in Table 3, in which the control mix of the polymers was named as Mix ID. The latex-to-cement ratio and the aggregate-to-paste ratio of the samples were kept constant at 0.41 and 3.33. The needed materials were weighed, the aggregate and cement were mixed in a mechanical mixer, and finally, the latex was added gradually during the progress of mixing before casting. All of the samples were prepared according to the Chinese Standard GB/T 50081-2019 [35], demolded after 24 h, and placed in a fog room at (20 ± 2) °C and 95% relative humidity for 28 days.

### 2.3. Test Methods

In this research, CMPC’s toughness is presented with compressive strength, flexural strength, flexural stiffness, impact resistance, and fracture toughness, so that the cube specimens, beam specimens, and cylindrical specimens were prepared for the corresponding test. Each test was carried out in 11 scenarios of the additive level for copolymer latex. The number of specimens for each scenario was six for the compressive strength test, three for the flexural strength test, three for the flexural stiffness test, five for the impact resistance test, and three for the fracture toughness test. The total number of specimens was 220. The specimens’ sizes and numbers are detailed in Table 4.

**Figure 2:**
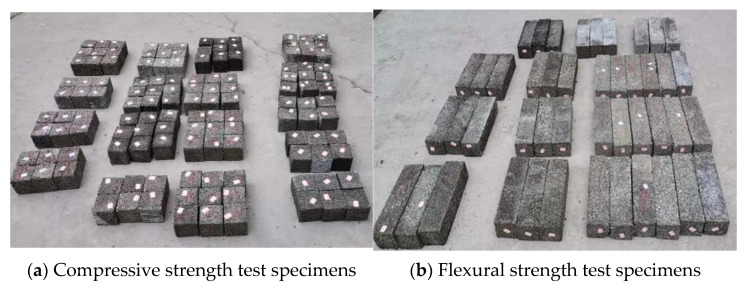

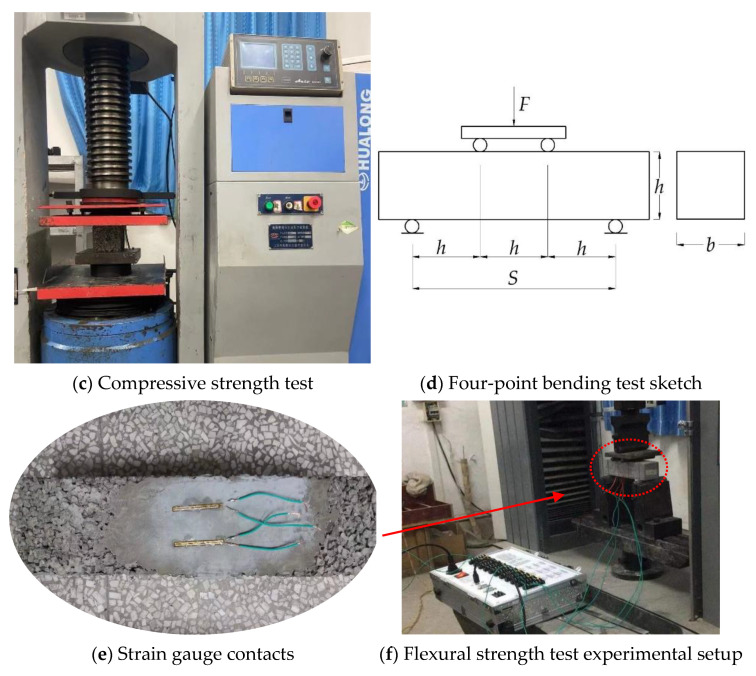
Compressive and flexural strength test schemes.

Compressive strength test and flexural strength test: The compressive strength and flexural strength were tested by following the Chinese Standard GB/T 50081 [35]. The cube specimens and beam specimens cured for 28 days were prepared as in Figure 2a,b. The compressive strength tests were conducted on a WHY-3000 microcomputer-controlled automatic compression testing machine, as shown in Figure 2c. The flexural strength of the specimen was measured by the four-point bending test method, as shown in Figure 2d. Two strain gauges aiming to detect the ultimate flexural strain were attached to the middle section of the specimen’s bottom, as shown in Figure 2e. Its sensing signal was transmitted by cables to the High-Speed Static Strain Data Acquisition and Analysis System, as shown in Figure 2f. The flexural strength was tested on a CMT5504 microcomputer-controlled electronic universal testing machine and was calculated as follows:

(1) $$\sigma_{f}=\frac{FS}{bh^{2}},$$

where $\sigma_{f}$ is the flexural strength (MPa), F is the rupture load, S is the distance between the supports (300 mm), *b* is the width of the specimen (100 mm), and *h* is the height of the specimen (100 mm).

**Figure 3:**
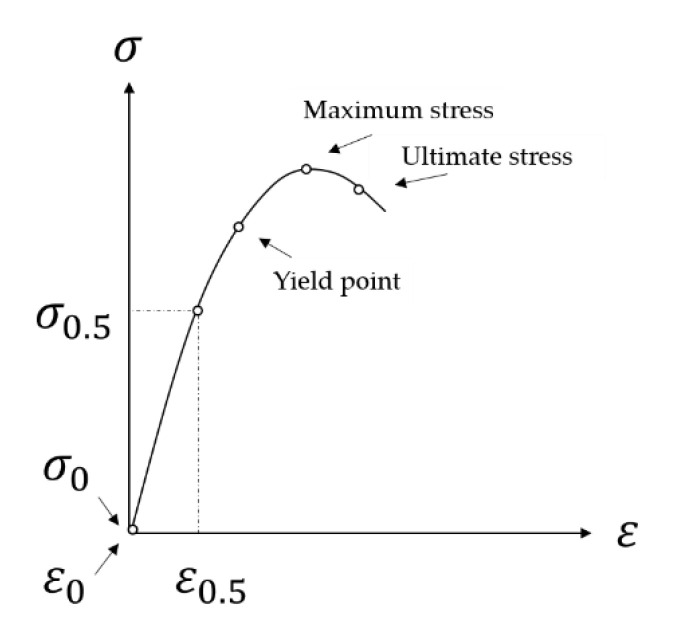
Bending stress–strain schematic.

Flexural stiffness test: The flexural stiffness revealed the stiffness measured in bend for characterizing the property of concrete pavement on orthotropic steel decks or subgrade [42]. The flexural stiffness was defined as the slope line that connected the origin and yield point by means of a bending stress–strain schematic method according to the literature [43,44], as shown in Figure 3. Nevertheless, the yield point is hard to detect so that the stress and strain under half of the rupture load, namely, $\sigma_{0.5}\;\mathrm{and}\;\varepsilon_{0.5}$ in Figure 3, are adopted in this study according to Chinese Standard JTG 3420 [45].

**Figure 4:**
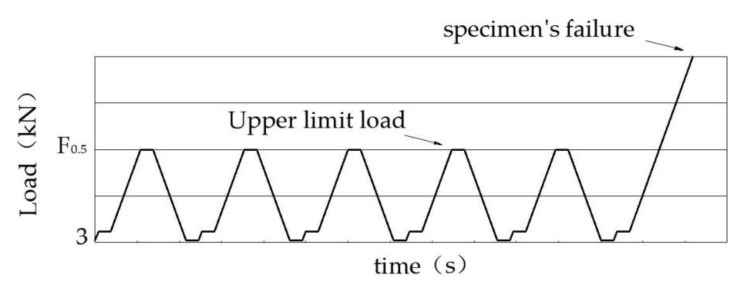
Loading cycle schematic.

The flexural stiffness of the specimens was tested at 28 days. Its test procedure was roughly similar to the flexural strength test; the only difference was the loading manner. The loading process was undertaken for five cycles as shown in Figure 4, in which the upper limit load was half of the rupture load, which was determined from the flexural strength test. The flexural strain was also detected by the strain gauges and High-Speed Static Strain Data Acquisition and Analysis System, as shown in Figure 2f. The flexural stiffness could be calculated by the following equations:

(2) $$E=\frac{∆\sigma }{∆\varepsilon }=\frac{\sigma_{0.5}-\sigma_{0}}{\varepsilon_{0.5}-\varepsilon_{0}}=\frac{\left(F_{0.5}-F_{0}\right)L}{bh^{2}\left(\varepsilon_{0.5}-\varepsilon_{0}\right)}\times 10^{3},$$

where E is the flexural stiffness at 28 days (MPa), ∆σ and ∆ε represent the flexural stress and strain increment of the specimen when loading from the initial state to the upper limit load. $\sigma_{0.5}$ and $\varepsilon_{0.5}$ are the specimen’s flexural stress and strain subjected to half of the rupture load (με), $\sigma_{0}$ and $\varepsilon_{0}$ are the specimen’s initial flexural stress and strain (με), $F_{0.5}$ is the upper limit load (kN), $F_{0}$ is the initial load (3 kN), L is the distance between the supports (300 mm), *h* is the height of the specimen (100 mm), and *b* is the width of the specimen (100 mm).

**Figure 5:**
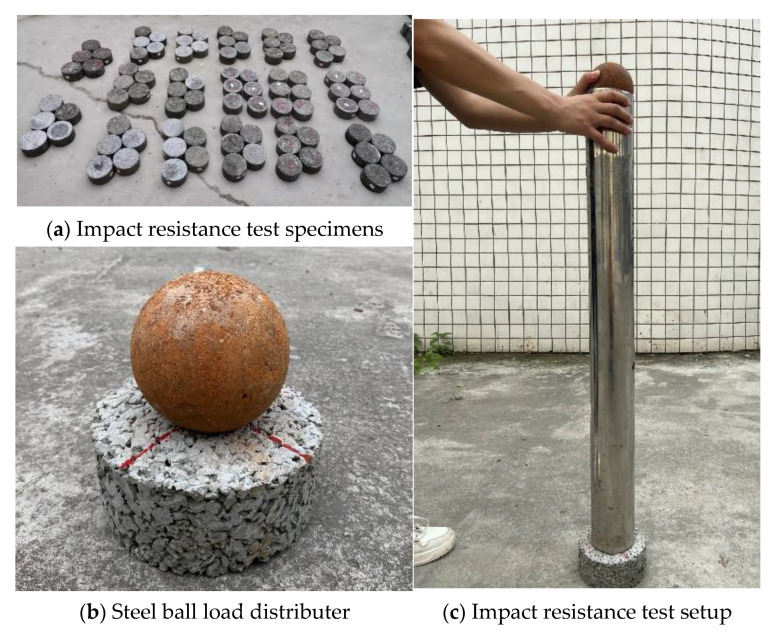
Impact resistance testing apparatus.

Impact resistance test: Impact resistance represents the CMPC’s energy absorption capability under repeated impact load. The impact resistance was tested at 28 days by using the Repeated Blows Drop Weight Impact (RBDWI) test according to American standard ACI 544-2R [32]. The blow numbers of the specimen’s first cracking and failure were considered as impact resistance; thus, the specimen’s absorbed impact energy could be calculated. In this research, modifications were suggested to simplify the test procedures. One steel ball with a 160 mm diameter and a 3 kg mass was used as a load distributor, while another steel ball with the same diameter and mass was used as a drop-weight and was dropped from the distance of approximately 690 mm to agree with the standard ACI 544-2R in terms of impact energy. The specimens were prepared as shown in Figure 5a. It was necessary to horizontally lay the specimen on the ground and locate the load-distributing ball on the central point of the specimen’s upper surface during the test, as shown in Figure 5b. A cylinder was used to fix the location of the load-distributing ball and dropping ball instead of the steel holding frame and steel holding ring so that the drop weight could drop freely on the top of the cylinder and directly impact the load-distributing ball, as shown in Figure 5c. The blow numbers were recorded, where *N**_1_*** denotes the first cracking number of blows, and *N*_2_ is the failure number of blows. The impact energy is calculated as follows:

(3) W=Nmgh,

where *W* is the absorbed impact energy by specimen, *N* is the number of blows, *m* is the drop weight (3 kg), g is the gravity acceleration (9.8 m/s^2^), and *h* is the drop distance (0.69 m).

**Figure 6:**
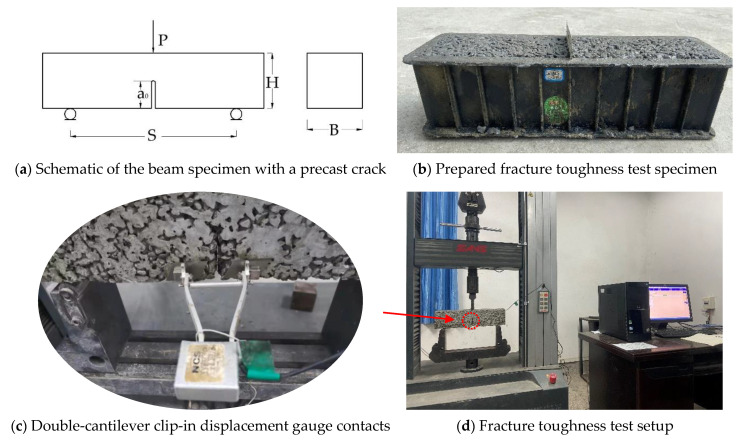
Fracture toughness test illustration.

Fracture toughness test: Pervious concrete’s fracture toughness is a measure of its ability to resist the unstable macroscopic crack growth. The most problematic concrete property is its response to the tensile load as the tensile strength; thus, the fracture mode I among all the fracture mechanics parameters was studied in this research [46]. The fracture toughness test was conducted according to Chinese Standard DLT5332-2005 [34] and the literature [47,48]: L = 400 mm, B = 100 mm, H = 100 mm, S = 300 mm, $a_{0}=50$ mm, as shown in Figure 6a. A beam specimen with one precast crack in the centerline of its bottom (as shown in Figure 6b) was used for three-point bending tests on a microcomputer-controlled electronic universal testing machine. The razor blades were positively attached adjacent to the precast crack, and the double-cantilever clip-in displacement gauge was recommended to detect the crack mouth opening displacement (CMOD), as shown in Figure 6c. The maximum loading capacity of the test machine was 50 kN, and the loading speed was 0.1 mm/min. The beam deflection is represented by the line displacement. The load–line displacement curve (P−L curve) and load–crack mouth opening displacement curve (P−CMOD curve) were recorded by the connected computer.

The fracture energy, $G_{F}$ (N·m), was calculated as follows [49]:

(4) $$G_{F}=\frac{w_{0}+mgv_{0}}{A},$$

where $w_{0}$ is the energy calculated from the area under the P-L curve, m is the specimen mass between the supports (6.6 Kg), g is the acceleration due to gravity, $v_{0}$ is the maximum deflection of the specimen, and *A* is the area of the fracture ligament, $A=B\times \left(H-a_{0}\right)$.

The fracture toughness $K_{IC}$ (kN/m^3/2^) was calculated as follows [47,48]:

(5) $$K_{IC}=\frac{\mathrm{PS}}{BH^{3/2}}\left[2.9\sqrt{\frac{a_{0}}{H}}-4.6\sqrt{{\left(\frac{a_{0}}{H}\right)}^{3}}+21.8\sqrt{{\left(\frac{a_{0}}{H}\right)}^{5}}-37.6\sqrt{{\left(\frac{a_{0}}{H}\right)}^{7}}+38.7\sqrt{{\left(\frac{a_{0}}{H}\right)}^{9}}\right],$$

where P is the rupture load (kN), S is the support span of the beam (300 mm), B is the thickness of the beam (100 mm), *H* is the height of the beam, and $a_{0}$ is the precast crack original depth (50 mm).

## 3. Results

### 3.1. Compressive Strength

**Figure 7:**
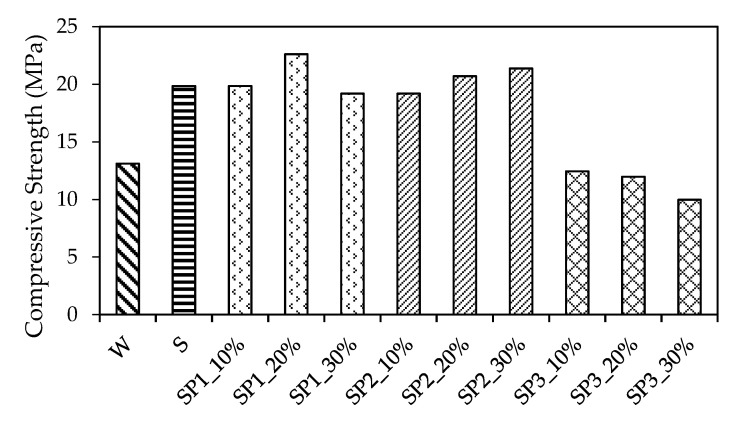
Comparison of compressive test results.

The compressive strength test results were determined and multiplied by the size conversion factor 0.95 according to the Chinese Standard GB/T 50081-2019 [35]. Figure 7 shows an overview of the results, in which pervious concrete with different polymer latex additives was assigned with different patterns. It was evident that the polymer and copolymer had a significant effect on the pervious concrete. First, SBR provided an increase in the compressive strength, which rose to 19.9 MPa. When the P1 was added into the SBR polymer latex, the results ranged from 19.2 to 22.6, in which SP1_20% (namely, proportion of P1 vs. SBR = 20% vs. 80%) exhibited higher values. The addition of P2 caused the compressive strength range from 19.2 to 21.4, in which the performance of pervious concrete increased with the increase in the P2 mix proportion. On average, the compressive strength of polymerized pervious concrete with respect to additive SBR, copolymer SP1 (namely, SP1_10%, SP1_20%, and SP1_30%), and copolymer SP2 (namely, SP2_10%, SP2_20%, and SP2_30%) rose to 19.9 MPa, 20.6 MPa, and 20.4 MPa, respectively. In other words, the growth rate of compressive strength was 151%, 157%, and 156%, respectively, after mixing with the polymer and copolymer latex in the pervious concrete, which indicated that the copolymer SP1 and SP2 enhanced the compressive resistance of pervious concrete greatly.

However, it could be seen that the CMPC with respect to SP3 failed to grow in terms of compressive strength (11.5 MPa in average); it decreased by roughly 13% compared with the non-polymerized pervious concrete. Above all, it could be observed that the polymer SBR, copolymer SP1, and copolymer SP2 could promote the compressive strength of the pervious concrete even though their enhancement effect on compressive strength was equal.

### 3.2. Flexural Strength

**Figure 8:**
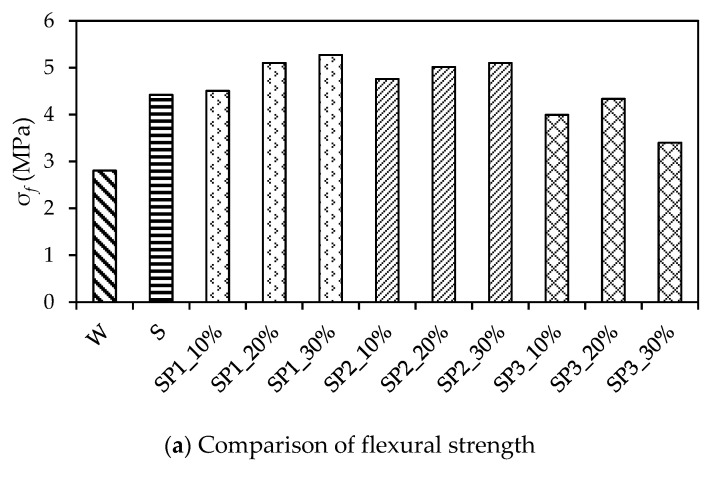

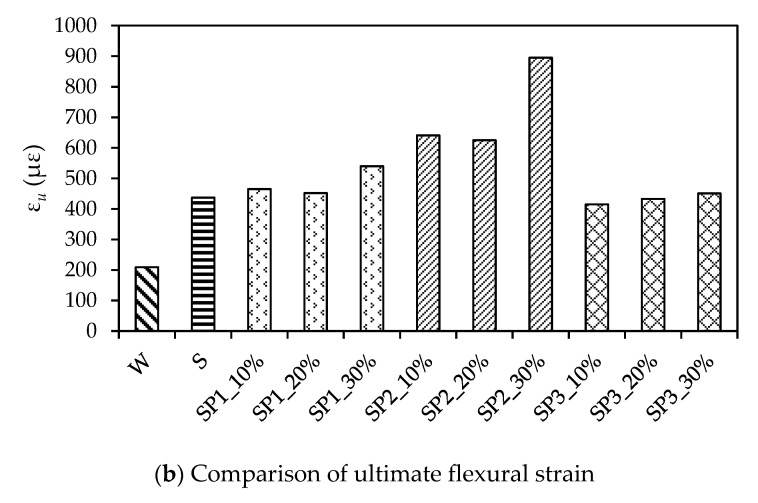
Comparison of flexural tests results.

The flexural strength test results were taken and multiplied by the size conversion factor 0.85 according to Chinese Standard GB/T 50081-2019 [35] as well. Figure 8a,b respectively presents the experimental data of $\sigma_{f}$ (flexural strength) and $\varepsilon_{u}\;$(ultimate flexural strain). It is apparent that the pervious concrete mixed with SBR, copolymer SP1, or copolymer SP2 obtained growth in both $\sigma_{f}$ and $\varepsilon_{u}$.

In terms of $\sigma_{f}$, the polymer SBR increased the test data to 4.4 MPa compared with the non-polymerized pervious concrete, with $\sigma_{f}$ 2.8 MPa, in which the growth rate was 58%. The copolymer SP1, SP2, and SP3 increased the average test data to 5.0 MPa, 5.8 MPa, and 4.6 MPa, respectively, in which the average growth rate of $\sigma_{f}$ was 77%, 107%, and 64%, respectively. Meanwhile, SBR increased $\varepsilon_{u}$ from 209 to 437, in which the growth rate of $\varepsilon_{u}$ was 110%; the growth rate of $\varepsilon_{u}$ for SP1, SP2, and SP3 was 130%, 240%, and 110%, respectively. It is evident that the $\sigma_{f}$ and $\varepsilon_{u}$ values of the pervious concrete were largely enhanced by SP1 and SP2 in contrast to the SBR, but SP3′s enhancement was slight. Additionally, both the $\sigma_{f}$ and $\varepsilon_{u}$ grew with an increase in the mix proportion of P1 and P2, although SP1_20% and SP2_20% showed a slight fluctuance on $\varepsilon_{u}$.

### 3.3. Flexural Stiffness

**Figure 9:**
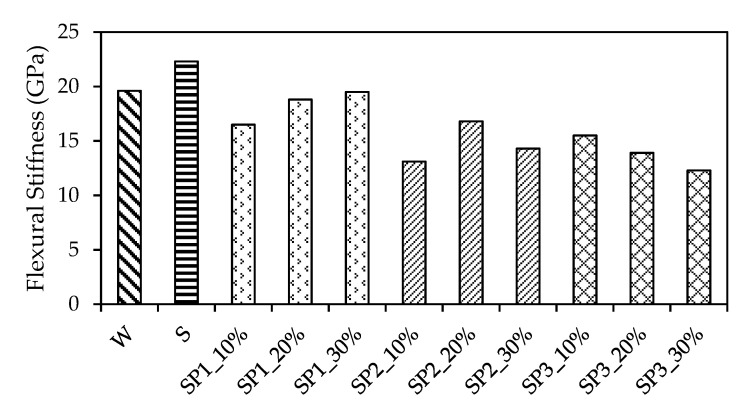
Comparison of flexural stiffness.

The *F_0.5_* and Δε during the flexural stiffness test are presented in Table 5. It is easy to observe that the trend of the change in Δε was different from that in *F_0.5_*. For example, the maximum *F_0.5_* in relation to SP1_30% did not induce a maximum Δε. The SP2_10% gave rise to a maximum Δε but not a maximum *F_0.5_*. Furthermore, the flexural stiffness test data were taken according to the Chinese Standard JTG 3420 [45] and are illustrated in Figure 9. It is obvious that the SBR led the flexural stiffness to a peak of 22.3 GPa. Then, the mean value of the flexural stiffness of the CMPC in relation to SP1, SP2, and SP3, which was 18.3 GPa, 14.7 GPa, and 13.9 GPa, showed a general decline along the mix ID axis. Additionally, a comparison within the test data in relation to SP1, SP2, and SP3 revealed that the copolymer differently affected the pervious concrete. First, SP1 increased the test data from 16.5 to 19.5 GPa, in which the value grew with an increase in the P1 mix proportion. Then, as to SP2, the test data rose from 13.1 to 16.8 GPa and then declined to 14.3 GPa. In the end, SP3 decreased the test data from 15.5 to 12.3 GPa, in which the test data declined with an increase in the P3 mix proportion. In general, the SP3 contributed to the lowest flexural stiffness, and the deformation performance of the pervious concrete significantly varied with the type and mix proportion of the polymer and copolymer additive even under the regular loading case.

### 3.4. Impact Resistance

**Figure 10:**
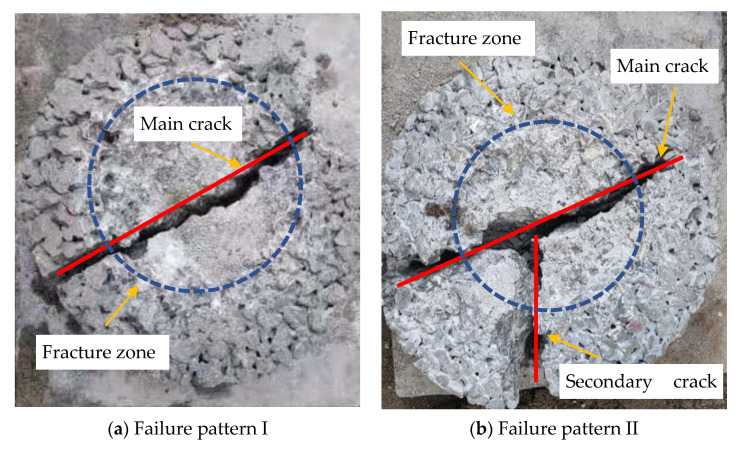
Failure patterns of the CMPC cylindrical specimens.

The impact resistance test results were taken according to American standard ACI 544-2R [32], and the two main ultimate fracture patterns of specimens are shown in Figure 10. It can be seen that the impacts led to the surface fracture in the central zone beneath the load-distributing ball. In Figure 10a, which illustrates failure pattern I, one top-to-down crack with a wide opening distance directly extended across the failure specimen, and the crack number stopped growing while the blows continued. This showed that some specimens’ failure came with the first visible crack, and that only blow numbers of *N*_1_ existed in the corresponding material. In Figure 10b, which illustrates the failure pattern II, the main crack with a wide opening distance also directly extended across the specimen, and the second crack starting from the center of the specimen extended to the same extent as the main crack. It is apparent that the corresponding material could bear more impacts after its first cracking. In addition, from both of the failure patterns, the materials can be seen splitting in a brittle manner owing to the cracks’ top-to-down, wide opening-distance, and roughly straight characteristics.

**Figure 11:**
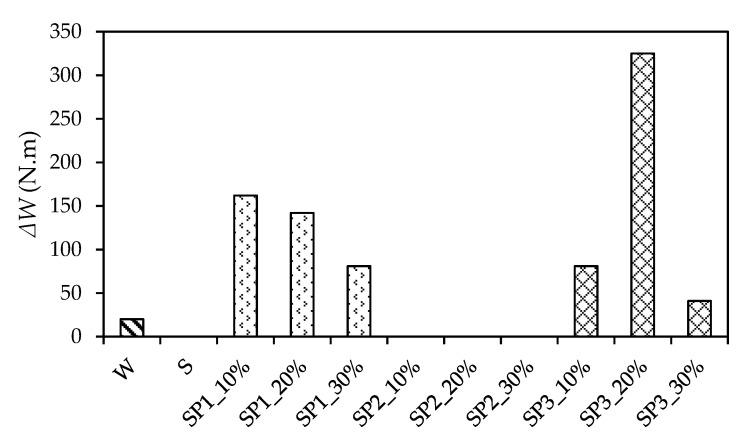
Comparison of ΔW.

Table 6 and Figure 11 presents an overview of the cracking number of blows, *N*_1_, and the failure number of blows, *N*_2_. The absorbed impact energy of the specimen accompanied with the first cracking is denoted as W_1_, and the further absorbed impact energy during the period from the first cracking to the specimens’ failure is denoted as ΔW, where $∆W=\left(N_{2}-N_{1}\right)\;\mathrm{mgh}$.

As shown in Table 6, the polymer and copolymer largely increased *N*_1_, the average of which was 629 for SBR, 872 for SP1, 1318 for SP2, and 164 for SP3. It is apparent that with the exception to SP3, the copolymer additive significantly increased the first cracking number compared to the SBR. Interestingly, *N*_1_ decreased with an increase in the mix proportion of P1 or P2. This implies that small quantities of P1 or P2 additive can easily enhance the pervious concrete’s impact energy absorption capability.

In addition, only slight differences between *N*_1_ and *N*_2_ were observed in Table 6. The blows from the first cracking to failure only occurred in relation to SP1 and SP3 apart from the non-polymerized pervious concrete. This can be easily observed in Figure 11, in which the CMPC with SP1 and SP3 was capable of absorbing an average of 128 N·m energy and 149 N·m energy after the first cracking. Furthermore, the ΔW decreased with the increase in the mix proportion of P1, which was similar to the W_1_ trend as shown in Table 6. Interestingly, the maximum ΔW occurred when the mix proportion of P3 was 20%. This showed that the CMPC with SP3 (especially SP3_20%) could absorb more energy after the first cracking.

Generally, it can be seen from Figure 11 that even if the CMPC with SP1 and SP3 could bear more impact blows after the first cracking, the CMPC’s brittle failure property was significant owing to the small quantity of ΔW. Considering the ultimate absorbed impact energy, which was obtained by adding W_1_ and ΔW, copolymer SP2 could mainly increase the CMPC’s impact resistance; its proper mix proportion was 10%.

### 3.5. Fracture Toughness

**Figure 12:**
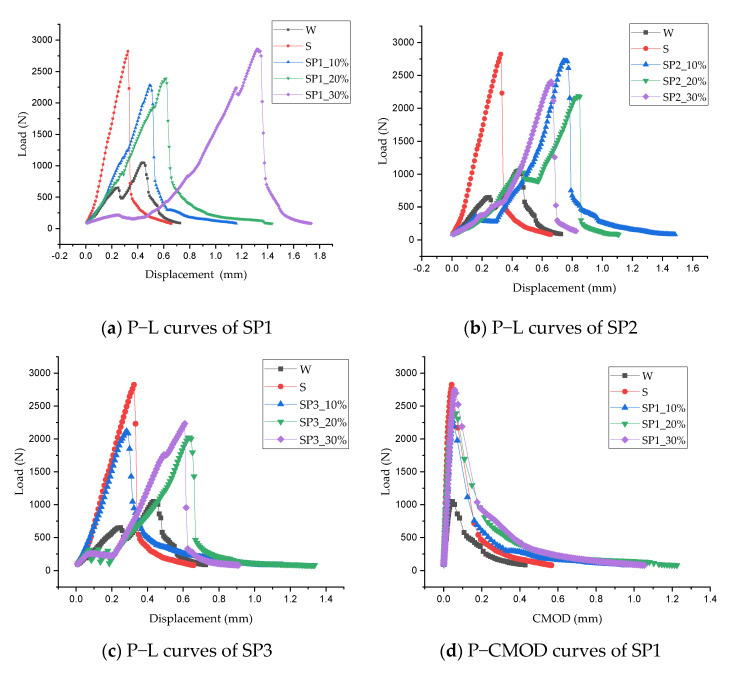

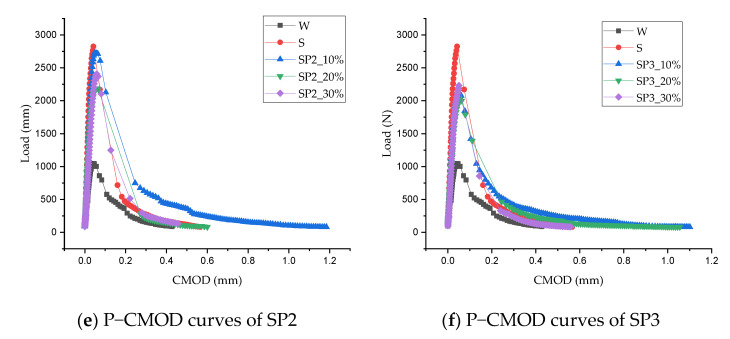
P−L and P−CMOD curves of the CMPC.

**Figure 13:**
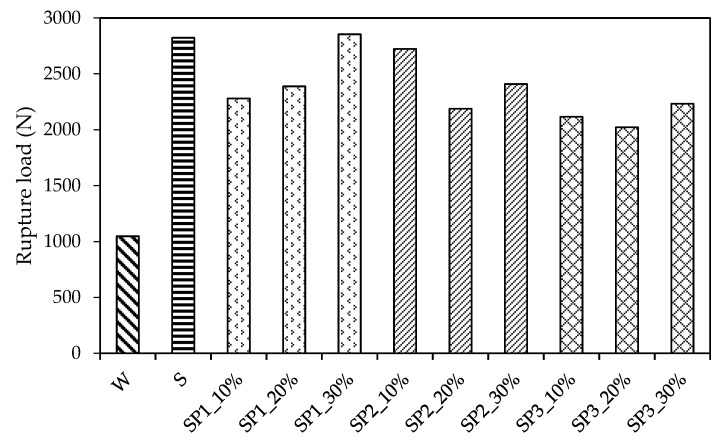
Rupture load of the fracture toughness test.

The fracture toughness test results were taken according to Chinese Standard DLT5332-2005 [34] and the literature [47,48]. The test data were analyzed qualitatively, as shown in Figure 12 and Figure 13, and several issues were identified: (1) strain-hardening behavior was observed (Figure 12a–c) when the beam deflection was 1.2 mm for SP1_30%, 0.2 mm for SP2_10%, 0.5 mm for SP2_20%, 0.1 mm for SP3_20%, and 0.1 mm for SP3_30%. The most likely reason is that polymer additives enhanced the materials’ resistance to the crack propagation. The reason for strain-hardening behavior occurring in non-polymerized pervious concrete needs to be further investigated; (2) the crack mouth opening distance had the same trend as that seen in Figure 12d–e. It is evident that the crack mouth opened slowly before achieving the rupture load, opened rapidly after achieving it, and finally, the opening speed remained constant until the specimen’s failure; (3) all of the materials exhibited a brittle failure observed from the P−L curves and P−CMOD curves; (4) as shown in Figure 13, the SP1_30% additive contributed the maximum value of 2855 N for the rupture load, which was followed by SBR and SP2_10% with 2823 N and 2723 N, respectively. The others were lower than 2408 N. All this indicates that the copolymer additives can largely enhance the pervious concrete’s capability to resist the fracture failure.

Table 7 presents the summary statistics for the effects of copolymer additives on the fracture energy and fracture toughness of the materials. In terms of *G_F_*, the SBR additive increased the test data from 737.9 to 1220.5 N·m. However, more than the SBR additive, the average value for the SP1 additive increased by 67.1%; for SP2, it increased by 40.7%, and for SP3, it increased by 17.2%. This indicated that the SP1 contributes greatly to the fracture energy absorption, and SP1_30% is the best performer, while SP2_10% is a close follower.

From the perspective of *K_IC_*, a slight increase from 347.6 to 360.3 kN/m^3/2^ was observed when the water was replaced by SBR latex. In contrast, significant increases were exhibited while the additives changed to copolymers. The average value for SP1 additive increased by 66.7%; for SP2, it increased by 78.2%; and for SP3, it increased by 59.4%. From the results, it is evident that when it comes to fracture toughness, SP2 plays an important role, and SP2_10% is the best performer, with the close followers being SP1_30% and SP3_10%.

In addition, another trend was observed: the *G_F_* and *K_IC_* grew with an increase in the P1 additive, while the P2 additive exerted opposite effects. This implies that a little mix of P2 can significantly increase pervious concrete’s ability to resist unstable macroscopic crack growth.

## 4. Discussion

### 4.1. Comprehensive Effect of Copolymer on Toughness of CMPC

From the previous section’s study, the compressive strength, flexural strength, ultimate strain, flexural stiffness, ultimate absorbed impact energy, fracture energy, and fracture toughness were selected as the toughness discriminating criteria. It is clear that the copolymer largely influences the CMPC’s toughness. However, it is difficult to determine which type of copolymer and which mix proportion of P1 or P2 is appropriate. Therefore, the following paragraphs aimed to identify the best performer that greatly increased the CMPC’s toughness. Data normalization was adopted to scale down the selected criteria to a range between 0 and 1. Then, the comprehensive effect of copolymers on CMPC could be determined by accumulating the normalized criteria value.

The data’s normalization process is illustrated in the following equation:

(6) $$D_{n}=\frac{D-D_{min}}{D_{max}-D_{min}},$$

where *D* is the original value of the test data,$\;D_{max}$ is the maximum value of the test data, $D_{min}$ is the minimum value of the test data, and $D_{n}$ is the normalized value of the test data.

**Figure 14:**
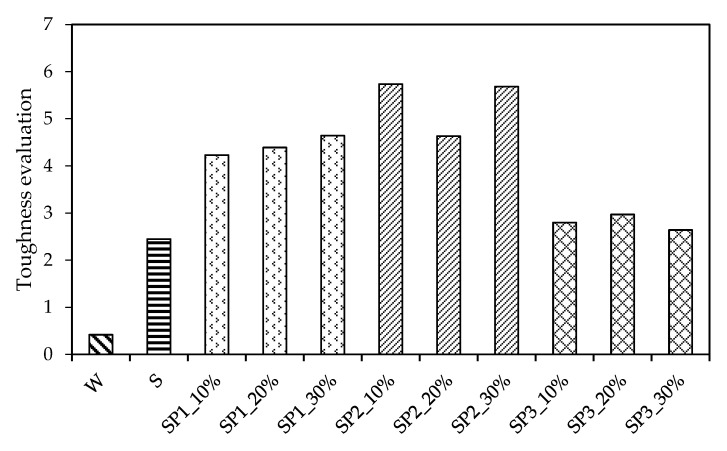
Comprehensive evaluation of effects of copolymers on toughness.

It is necessary to take the reciprocal of the flexural stiffness before normalizing it, as the flexural stiffness is inversely proportional to flexural toughness. The comprehensive effect of copolymers on CMPC’s toughness is illustrated in Figure 14. It is apparent from the figure that the polymer additives largely increased the toughness of the pervious concrete. The SBR additive increased the comprehensive data from 0.42 to 2.45. The comprehensive data for SP1, SP2, and SP3 were, on average, 4.42, 5.35, and 2.8, respectively. This indicates that the P2 additive mainly enhances the toughness. Furthermore, SP2_10% led to the highest comprehensive data, which can be seen in the Figure 14. Thus, it can be concluded that the copolymer with a little proportion of XG–6001 acrylate polymer latex (e.g., 10%) has a strong potential to prevent the pervious concrete from fracture while improving the pervious concrete’s deformation capability.

The reason for most of the copolymer additives in this study increasing the mechanical properties can be explained as follows: the plain cement paste coating the aggregate has high fluidity compared to the copolymerized cement paste during the specimen cast molding period. Considering the relatively high polymer-to-cement ratio in this study and the copolymer’s potential ability of enhancing the adhesion of cement paste, the copolymerized cement paste was more likely adhere to the aggregate surface, while the plain cement paste tended to flow to the lower place. This caused a strong bonding between the aggregates of the CMPC while a weak bonding existed in the plain pervious concrete; thus, the relative excellent mechanical properties of the CMPC was induced.

Another point that needs to be mentioned is that in previous studies [12,14], the PMPC without sand had a 28-day compressive strength of 11.29 MPa, and even most of the PMPC specimens with less sand had poor compressive strength. Interestingly, this study demonstrated that most of the CMPC specimens were of higher compressive strength than 15 MPa, as shown in Figure 7. This verified that the emulsion copolymerization method is capable of enhancing the bonding between aggregates and indicated that the proposed CMPC is of relative high stability and durability in contrast with the previous studies.

In addition, according to the Chinese standard JTG D40-2011 [50], the concrete with flexural strength between 4.5 and 5.0 MPa could be used to construct the pavement subjected to medium traffic load. Apparently, the CMPC in relation to SP2_10% in this research is applicable in traffic road construction owing to its average flexural strength of 4.8 MPa, as shown in Figure 8. The CMPC with other mix proportions of SP1 or SP2 could be subjected to a heavy or extremely heavy traffic load owing to their average flexural strength of more than 5.0 MPa.

### 4.2. Toxicity Evaluation of CMPC

The SBR polymer was proved to be non-toxic in the literature [51]; therefore, the acrylate polymers needed to have a toxicity evaluation to estimate their impact on the environment in terms of chemical safety. Since the optimal acrylate type is XG–6001 acrylate polymer, thus, it was sent to have a toxic test in SGS-CSTC standards technical services (Shanghai) CO., Ltd., Shanghai, China. Its toxic test data are shown in Table 8, where the tested toxicity indicators are listed in the “Items” column. While the tested item falls in the range of limits, it means the corresponding toxicity indicator meets the environment friendliness requirements, and thus, the “N/Y” column will be noted as “Y” or vice versa. It can be concluded from the table that all the toxic test results fall in the range of limits and the XG–6001 acrylate polymer meets the chemical safety environmental system requirements. This implies that the CMPC with single-sized aggregates and no sand is a promising alternative in sustainability traffic road construction.

## 5. Conclusions

This study investigated the toughness of SBR acrylate polymer-modified pervious concrete with single-sized aggregates and no sand. In addition, the optimal acrylate type and its mix proportion for the CMPC without sand were proposed. The following conclusions were drawn from the results:

(1) The combination of SBR and acrylate polymer largely increases the toughness of pervious concrete without sand, indicating that the reduction of the sand mixture is feasible while maintaining the pervious concrete’s mechanical performance.

(2) The toughness of CMPC is largely affected by the type and mix proportion of acrylate polymer. Furthermore, the influence trend varies according to different mechanical indexes. The normalization process of test data, which scales the data with different dimensions down to the range from zero to one, is applicable to comprehensively evaluate the effect of a copolymer on the toughness of CMPC.

(3) XG–6001 acrylate polymer mainly and comprehensively enhanced the toughness of the CMPC in contrast with other acrylate polymers. It was difficult to improve the enhancing property of the XG–6001 acrylate polymer with the growth of its mix proportion.

(4) The zero-sand pervious concrete with 90% SBR and 10% XG–6001 acrylate emulsion copolymerization exhibits relatively high toughness compared with previous studies in the literature, demonstrating its ability to retain stability, durability, and cracking resistance ability, and proving its strong application potential in sustainability traffic road construction.

In this study, the polymer-to-cement ratio was relatively high, and the testing program and statistical evaluation applied only investigated the toughness of the CMPC in a common environment; thus, the further study will seek to optimize the polymer-to-cement ratio and pay attention to the performance of CMPC in special conditions, such as freeze–thaw and erosion. Enhancing the skid resistance for the CMPC while retaining its high toughness property is an area of further study as well. Micro-surfacing technology is a potential way to promote the skid resistance of the CMPC in the field in terms of enriching its micro-texture based on its macro-texture. The authors will investigate the probable specific micro-surfacing manners or more effective methods to raise the traffic safety of CMPC pavement in further researches.

## Figures and Tables

**Table 1:** Composition of the used Portland cement (wt %).

SiO_2_	Fe_2_O_3_	Al_2_O_3_	CaO	MgO	SiO_3_	Analysis (%)
22.4	3.15	5.6	59.58	2.58	2.42	Cement

**Table 2:** Polymer latex characteristics.

	SBR	P1	P2	P3
Chemical family	Styrene–butadiene rubber	XG–6161 acrylate	XG–6001 acrylate	XG–2135 acrylate
Solid content, wt %	45 ± 1	56 ± 1	55 ± 1	50 ± 1
PH	7 to 8.5	6.5 to 8	7 to 9	7 to 9
Density [kg/m^3^]	1.20 to 1.22	1.08 to 1.10	1.10 to 1.12	1.18 to 1.20
Viscosity [MPa.s]	1500 to 2500	500 to 1500	1000 to 2500	800 to 2500
Appearance	Milky white liquid emulsion	Milky white and grayish liquid emulsion	Milky white and grayish liquid emulsion	Milky white and grayish liquid emulsion

**Table 3:** Mix proportion of copolymer latex.

Control Mixes	Mix ID	Control Mixes	Mix ID
100% Water	W	80% SBR + 20% P2	SP2_20%
100% SBR	S	70% SBR + 30%P2	SP2_30%
90% SBR + 10% P1	SP1_10%	90% SBR + 10% P3	SP3_10%
80% SBR + 20% P1	SP1_20%	80% SBR + 20%P3	SP3_20%
70% SBR + 30% P1	SP1_30%	70% SBR + 30% P3	SP3_30%
90% SBR + 10% P2	SP2_10%		

Notes: 100% water means pervious concrete without polymer latex, while 100% SBR means without additives. Control mixes for other copolymers are based on the SBR-to-additive ratio (i.e., 90% SBR + 10% P1 means the SBR’s portion in the latex was 90%, while P1′s portion was apparently 10%).

**Table 4:** Specific size and number of the specimens.

Test Type	Specimen Size [mm]	Number of Scenarios	Number of Specimens
Compressive strength	100 × 100 × 100	11	66
Flexural strength	100 × 100 × 400	11	33
Flexural stiffness	100 × 100 × 400	11	33
Impact resistance	Diameter 152, height 63.5	11	55
Fracture toughness	100 × 100 × 400	11	33

**Table 5:** Test data of upper limit load and flexural strain increment.

Mix ID	*F_0.5_* (KN)	∆ε (με)
W	5.5	30.67
S	8.65	67.3
SP1_10%	8.85	109.25
SP1_20%	10	95.6
SP1_30%	10.35	107.8
SP2_10%	9.35	161.4
SP2_20%	9.85	125
SP2_30%	10	146.7
SP3_10%	7.85	76.4
SP3_20%	8.5	129.9
SP3_30%	6.65	109.4

**Table 6:** Test data of Impact Resistance.

Mix ID	*N*_1_	*N*_2_	W_1_
W	55	56	1136
S	629	629	12,760
SP1_10%	1250	1258	25,520
SP1_20%	811	818	16,594
SP1_30%	555	559	11,340
SP2_10%	1645	1645	33,371
SP2_20%	1020	1020	20,692
SP2_30%	1289	1289	26,149
SP3_10%	126	130	2637
SP3_20%	307	323	6552
SP3_30%	58	60	1217

**Table 7:** Fracture energy vs. fracture toughness.

Mix ID	*G_F_* (N·m)	*K_IC_* (kN/m^3/2^)
W	737.9	347.6
S	1220.5	360.3
SP1_10%	1663.2	549.9
SP1_20%	1972.6	556.2
SP1_30%	2483.7	632
SP2_10%	1991.8	651
SP2_20%	1569.8	587.8
SP2_30%	1590.6	619.4
SP3_10%	1252.9	632
SP3_20%	1565.7	493
SP3_30%	1469.7	537.2

**Table 8:** Toxic test results of the XG–6001 acrylate polymer.

Items	Limits	Unit	Test Value	N/Y
Benzene	0.2	g/kg	0.1	Y
Toluene and xylene	10	g/kg	4	Y
Volatile organic chemicals (VOCS)	401	g/L	50	Y
Internal exposure index	1		0.1	Y
External exposure index	1		0.1	Y
Radium-226	0	Bq/kg	0	Y
Thorium-232	0	Bq/kg	0	Y
Potassium-40	0	Bq/kg	0	Y

## Data Availability

Data are contained within the article.
